# Modern health worries and exposure perceptions of individuals reporting varying levels of sensitivity to electromagnetic fields: results of two successive surveys

**DOI:** 10.3389/fpubh.2025.1536167

**Published:** 2025-02-19

**Authors:** Maryse Ledent, Benjamin Vatovez, Philippe Roelandt, Jimmy Bordarie, Maël Dieudonné, Els De Waegeneer, Caroline Kremer, Laura Boucher, Catherine Bouland, Eva Maria De Clercq

**Affiliations:** ^1^Chemical and Physical Health Risks, Sciensano, Brussels, Belgium; ^2^École de Santé Publique, Université Libre de Bruxelles, Brussels, Belgium; ^3^Cellule Champs Électromagnétiques, Institut Scientifique de Service Public (ISSeP), Liège, Belgium; ^4^Qualipsy (UR1901), University of Tours, Tours, France; ^5^Centre Max Weber, Institut des Sciences de l’Homme, Lyon, France; ^6^Department of Public Health and Primary Care, Ghent University, Ghent, Belgium

**Keywords:** idiopathic environmental intolerance attributed to electromagnetic fields (IEI-EMF), modern health worries (MHW), exposure perception, avoidance strategies, sensitivity

## Abstract

**Introduction:**

Individuals who claim to be affected by idiopathic environmental intolerance attributed to electromagnetic fields (EMFs) report symptoms linked to EMF exposure. Uncertainties about the causes of these symptoms often leave them seeking their own care solutions. In our connected societies, they may limit their exposure, leading to a spiral of avoidance that negatively impacts overall health. Our objective is to gain insights into the characteristics of people who report sensitivity to EMFs in an attempt to provide care guidance. This study focuses on modern health worries (MHW), behaviours, and exposure perceptions of people reporting various sensitivity levels to EMFs during the COVID-19 lockdowns, which altered habits and increased telecommunication device use.

**Methods:**

We conducted two surveys during relaxed lockdown periods in Belgium (June/July 2020 and February/March 2021). A total of 97 and 285 participants, respectively, answered a questionnaire on sensitivity to EMFs, MHW, exposure perception, and strategies to limit EMF exposure. We applied nonparametric descriptive and multivariate statistical analyses.

**Results:**

Higher sensitivity to EMFs correlates with greater MHW regarding EMF sources and more strategies to limit EMF exposure. However, these strategies were inconclusive, as many still felt highly exposed to EMFs.

**Discussion:**

Given the high distress, social isolation, and professional difficulties faced by some EMF sensitive individuals, the relevance of exposure avoidance strategies is questionable. People who perceive high sensitivity to EMFs report worries and avoidance behaviors, yet still feel highly exposed. The findings suggest exploring new care avenues.

## 1 Introduction

The context of the COVID-19 pandemic, with successive imposed lockdowns, has led the population to change certain habits and to intensify the use of mobile devices (1) and screens, particularly for browsing social networks and the Internet for daily life needs (shopping, banking, administration, etc.) (2). Furthermore, the pandemic coincides with the roll-out of the fifth generation (5G) of mobile networks in several countries, which, in the absence of clear evidence to draw conclusions (3), has led to uncertainty and misinformation about the impact of 5G on human health (4).

The perception of health risks related to modern technologies is widespread (5–8). This is referred to as Modern Health Worries (MHW) (8, 9) and includes electromagnetic fields (EMFs), air pollutants, food additives, overuse of antibiotics, noise, and other broad environmental issues, such as ozone layer depletion or climate change.

Environment-related syndromes, referred to as idiopathic environmental intolerances (IEIs) are polymorphic (e.g., sick building syndrome, sensitivity to multiple physical, chemical, and biological agents) (9, 10) and they affect a considerable number of people. Worldwide, in the absence of validated objective criteria, the reported IEI-EMF prevalence varies considerably from one survey to another, ranging from 1.5 to 13% (11). IEIs refer to syndromes in which patients describe symptoms related to environmental exposures, whereas there is no detectable abnormality on clinical examination (10). Among the physical agents, non-ionizing EMF are at the origin of the so-called IEI attributed to EMF (IEI-EMF) syndrome, also referred to as electrohypersensitivity (EHS). People reporting IEI-EMF (IEI-EMF people) claim to suffer from a variety of symptoms attributed to EMF sources in the absence of validated clinical or biological evidence (11, 12). The incriminated sources are diverse, including the entire non-ionizing radiation (NIR) part of the electromagnetic spectrum, covering extremely low frequencies - generated by electrical equipment and appliances - to radio frequencies (RF) - emitted by mobile phones, base stations, Wi-Fi, Bluetooth, etc.

Studies exploring the association between EMF exposure and reported IEI-EMF symptoms have failed to provide conclusive evidence. Indeed, Schmiedchen et al. (13) indicated that methodologically sound provocation studies pointed to an unlikely effect of EMF exposure. Given the ubiquity of man-made EMF sources in our societies, physical and psychological symptoms could also be the consequence of adverse expectations (whether conscious or not) of EMF exposure (14, 15). However, such nocebo effects should not be considered exclusive to other causes, as shown in several qualitative studies on the trajectories of IEI-EMF people showing that symptoms can be present before being attributed to EMF (16–18).

Regardless of the origin of the symptoms, the suffering of many of these people is very significant and leads into spirals of exposure avoidance strategies incompatible with overall health (19) and quality of life. In MHW, an association is repeatedly reported with symptoms, either directly (6, 20–22) or indirectly through, e.g., the number of visits to general practitioners (23) and IEI (5, 9). The results are similar for the IEI-EMF (24, 25). Awareness of this possible association may be informative for patient support in the healthcare system (21). In addition, beyond the evaluation of risk perception on the MHW scale, it appears essential to complement the analysis with exposure perception. Indeed, in IEI-EMF, coping strategies most often involve exposure avoidance (26) by taking various protection measures, such as switching off home RF sources, using protective solutions, and asking relatives to switch off mobile phones (16, 17). Some IEI-EMF people then report an improvement in their quality of life, while others do not, and go further with exposure avoidance strategies. This results in social exclusion, work incapacity and financial difficulties (11, 27), often reinforced by a lack of understanding of the precautions taken within their family and professional circles (11). The effectiveness of avoidance strategies can be questioned and analysed in relation to exposure perception. All the more so as the direction of the association between exposure perception and the question of avoidance strategies clearly appears to be extremely complex, as does the association between sensitivity and concern.

Our primary aim is to gain new knowledge about the characteristics of IEI-EMF people in an attempt to provide guidance for care. More specifically, in this work, we investigated the associations between EMF sensitivity and MHW, exposure perception and avoidance strategies. Three hypotheses are explored:

*Hypothesis 1*: The more people reported being EMF sensitive, the greater their worries, particularly on the EMF items of the MHW scale.

*Hypothesis 2*: The more people reported being EMF sensitive, the greater they adopted avoidance strategies.

*Hypothesis 3:* The more people adopt exposure avoidance strategies, the lower their perceived exposure to EMF sources.

## 2 Methods

### 2.1 Survey, recruitment and data collection

This study took place in two distinct periods, during the relaxation of lockdown conditions after the first and second waves of COVID-19 in Belgium, respectively, while the roll-out of 5G in Belgium was launched in April 2020, which led to numerous protests among the concerned citizens.

The survey was developed under.Net (C#) with storage in an internal Structured Query Language (SQL) server database. In the first period (P1), the survey was published online in French (June 2020) and in Dutch (July 2020) and was available online for 3 weeks. This period corresponds to the relaxation of the lockdown measures following the first COVID-19 wave. In the second period (P2), it was published in French (February 2021) and in Dutch (March 2021) and was available online for 4 weeks. This second period corresponds to the post-COVID-19 lockdown period, although less strict, linked to the second wave of COVID-19 (Table 1).

**Table 1:** Summary table of periods and total data collection.

	P1	P2
French language	June 2020 During 3 weeks	February 2021 During 4 weeks
Dutch language	July 2020 During 3 weeks	March 2021 During 4 weeks
Total respondents	153	446
Included participants	97	285
Recruitment	Direct contact with individuals who had been involved in previous projects on the IEI-EMF, either sensitive or not contact with regional institutions contact with associations representing the interests of people with EMF sensitivities and other environmental or health prevention associations researchers’ social networks	The same contacts were used information was also sent to new contacts, for the most part in Flanders information was also shared through highly followed institutional channels (to increase the visibility of the survey in social networks)

P1: Period 1; P2: Period 2.

Respondents who did not wish to complete the survey digitally could request a paper format to be filled in by hand and sent by post. The final anonymized dataset includes one hand-filled form in P1 and two in P2.

### 2.2 Questionnaire survey

The survey included the following domains (see details in Supplementary material S1).

*Demography* includes questions related to age, gender, municipality, and employment (possibly temporarily interrupted). The degree of urbanization (DEGURBA) was assigned based on the municipality (28). The classification identifies three zones (level 1): cities, towns and suburbs, and rural areas.

*Health status:* Respondents were asked to evaluate their health on a 5-point Likert scale (from very good to very poor—Health_status) and to report the frequency over time of symptoms common in the IEI-EMF (migraine, insomnia, fatigue, memory problems, heart palpitations, joint pain, digestion problems, itching, depressed mood, and irritability) in four categories (from never to every day). The symptom score was calculated by averaging the answers related to symptoms (SymptomScore).

*Risk Perception, by way of the MHW scale* (8, 20): The scale was translated into French and Dutch and used to assess how concerned respondents perceive the impact of various aspects of modern life on their health. Translations were independently proofread by two members of the research team in both languages. The scale initially consists of 24 items, with scores ranging from 0 (not at all concerned) to 4 (extremely concerned). Two items were added to consider new issues: risk perceptions linked to “5G antennas” and “COVID-like viruses,” while due to technical issues, the item on genetically modified food was removed (Supplementary material S2). MHW scores were calculated by summing the answers. This resulted in an overall MHW score (25 items), a specific score for items related to radiation (4 items: mobile phones, 2G-4G antennas, 5G antennas, high voltage powerlines (HVPL)—EMF_worries score) and an MHW score excluding radiation items (21 items—noEMF_worries score), ranging from 0 to 100, 0 to 16 and 0 to 84, respectively. The four items related to EMF sources were also considered separately, each ranging from 0 to 4 (mobile phone-worries, 2G-4G antennas-worries, 5G antennas-worries, and HVPL-worries).

*Exposure perception:* The level of exposure perception to the various agents of the MHW scale was evaluated by the following question: “Are you very exposed to this agent?” (yes/no). Exposure scores were calculated, assigning 1 to “yes” and 0 to “no” answers, resulting in two exposure scores ranging from 0 to 4 and 0 to 21 for items related to EMF (EMF_exposure) or not (noEMF_exposure), respectively. The perception of exposure to the four items on EMF sources was also considered one by one, ranging from 0 to 1.

*EMF sensitivity*: The respondents’ perceptions of their sensitivity to EMF were examined using five categories (from not sensitive to hypersensitive).

*Exposure avoidance strategies:* These strategies could be used to reduce exposure (Supplementary material S3). A score was calculated: for each strategy, 1 or 2 points were assigned if the strategies had been in place for less or more than 1 month, respectively, to give more weight to those strategies adopted over a longer period of time. An avoidance score was derived by summing the answers to the 15 questions, ranging from 0 to 30.

### 2.3 Statistical analyses

Univariate statistics were processed with Stata/SE 15.1. Fisher’s exact test was used to compare the characteristics of the participants between the two periods, as well as to compare the questionnaires included/excluded due to missing information on sensitivity to EMF and MHW. The distribution of results between the different sensitivity categories was also examined using this test.

Comparisons of health status and EMF sensitivity by period were performed by ANOVA. Comparisons related to EMF sensitivity were performed by ANOVA. The distribution of the results among the different categories of sensitivity was explored by the chi-squared test.

Multivariate analyses were conducted to integrate the contributions of the different variables to the varying levels of EMF sensitivity reported by respondents: (1) ordered logistic regression (OLR) with Stata/SE 15.1 and (2) exploratory multivariate analyses using gradient boosting machine (GBM) analysis within the R environment (R version 4.1.2, The R Foundation for Statistical Computing). GBM modeling is a machine learning technique of interest in managing possible non-linear relationships between independent and dependent variables, without requiring explicit model specifications. The boosting approach used in boosted regression trees has *its origins within machine learning (46), but subsequent developments in the statistical community reinterpret it as an advanced form of regression (45)* [(29), p.803]. As recommended by Elith et al. (29), we were able to fit GBM models with at least 1,000 trees. A Poisson distribution fits the EMF-sensitivity variable. The GBM prediction models were fitted following the gbm.step routine in the gbm package version 2.1.8 and dismo package version 1.3–5. The trees were built with default parameters: a tree complexity of 5, a learning rate of 0.001 and a bag fraction of 0.5. To enable analyses to be replicated, the seed was set at 123.

In both multivariate analyses, the dependent variable was EMF sensitivity, while the independent variables were gender, age, employment, region, and urbanization as generic variables; symptom score and health status as health variables; and MHW, exposure perception and avoidance scores as specific variables, based on our hypotheses.

### 2.4 Study samples

We used a convenience sampling method. In P1 and P2, 153 and 446 people, respectively, participated in the survey. However, as a number of them did not complete the questions on sensitivity to EMF and the MHW scale, they were excluded from further analysis. Thus 97 participants were included in P1 (57.5% female and 42.5% male) and 285 (52.6% female and 47.4% male) in P2 (see Supplementary material S4).

In P1, no significant differences were found between the general characteristics of the included and excluded participants (Supplementary material S4). In P2, the proportions of women (*p* = 0.037) were slightly higher in the excluded participants group.

In the included participant group, the respondents’ age category distribution, and sex distribution were similar between the two periods (Supplementary material S4). However, the distribution of living areas (Region variable) differed between the two periods (*p* < 0.001), with a higher proportion of Walloon residents in P1 and higher proportions of Brussels and Flemish residents in P2 due to the more intensive recruitment in those regions, especially in Flanders. The difference in the degree of urbanization distributions (*p* = 0.001) follows the regional characteristics, as indicated by the population density in the three regions of 488, 7,511, and 216 inhabitants/km^2^ in Flanders, Brussels and Wallonia, respectively (30). Approximately 70% of the respondents, in both periods, declare themselves professionally active.

Regarding health, EMF sensitivity, symptoms and (Table 2), 26.8% of the P1 respondents and 33.7% of the P2 respondents stated that they were not sensitive to EMF, 46.3% of the P1 respondents and 46% of the P2 respondents were not very sensitive or somewhat sensitive, while 26.8% of the P1 respondents and 19.6% of the P2 respondents stated that they were very sensitive or hypersensitive to EMF. There were no significant differences in the sensitivity distribution across the different categories between P1 and P2.

**Table 2:** Health, perceived EMF sensitivity and symptoms reported by participants: comparisons between included and excluded participants (due to missing data on sensitivity and MHW), and between P1 and P2 (included only).

	P1			P2			
	Included	Excluded	Inc/Exc	Included	Excluded	Inc/Exc	P1/P2 (included)
	*n* = 97	*n* = 56	*p*-value	*n* = 285	*n* = 161	*p*-value	*p*-value
Health status (%)							
Very good	7.5	22.7	0.037	18.1	20.4	0.531	0.434
Good	51.9	42.3		49.0	44.2		
Fair	35.2	23.7		22.8	28.1		
Poor	5.6	11.3		9.4	6.3		
Very poor	0	0		0.7	1.1		
Perceived EMF sensitivity (%)							
No sensitive	26.8	3.6	0.311	33.7	3.1	0.028	0.484
Not very sensitive	21.6	12.5		18.9	3.1		
Somewhat sensitive	24.7	5.4		27.7	5.0		
Very sensitive	16.5	1.8		11.9	5.0		
Hypersensitive	10.3	1.8		7.7	3.7		
(Missing)		75			80.1		
Symptom score							
Never to almost once a week	33.3	42.3	0.09	33.7	35.8	0.693	0.525
Once a week to almost every week	61.9	43.3		52.9	48.1		
Every week to every day (or almost)	4.8	14.4		13.5	16.1		

P1: Period 1; P2: Period 2; Inc/Exc: Included versus Excluded.

In P1, a higher proportion of people in the excluded participants group rated their health as very good. In P2, both the proportions of women (*p* = 0.037) and of the most sensitive individuals (*p* = 0.028) were slightly higher in the excluded group.

## 3 Results

### 3.1 Hyp 1: the more EMF sensitive people are, the greater the worries

**Figure 1:**
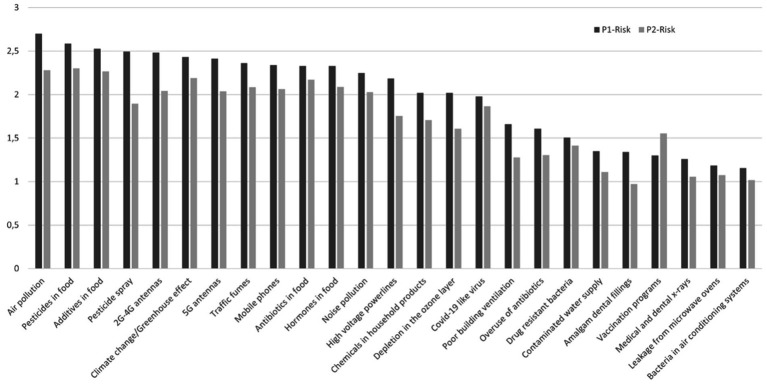
Histogram of MHW (from 0 “not at all concerned” to 4 “extreme concern”) in P1 and P2.

MHW assessment did not reveal an extreme concern, with the highest averages ranging from moderate to very much in P1 for air pollution, pesticides in food, additives in food, pesticide sprays and mobile phone antennas, and in P2 for pesticides in food, air pollution, additives in food, climate change and antibiotics in food (Figure 1). In relation to the COVID-19 pandemic-related variables, concerns are less than moderate.

The MHW and EMF worries showed significantly different values in the respective EMF sensitivity categories in the two periods: respondents who considered themselves more sensitive showed greater concern than those who considered themselves not sensitive or somewhat sensitive, both in terms of the MHW and EMF worries scores (Table 3), as well as in terms of the items related to EMF sources considered one by one (Table 4). Greater differences between the EMF sensitivity categories were observed for mobile phones and 2G-4G antennas, with mean scores ranging from less than 1 for not at all sensitive to 4 or close to 4 for extremely sensitive respondents.

**Table 3:** MHW and EMF_worries scores, by period (mean [95% CI]).

		P1	P2
Modern Health Worries			
EMF-sensitivity	Not at all	36.04 [28.8–43.28]	30.15 [25.9–34.39]
	Somewhat	48.14 [36.21–60.08]	44.22 [38.44–50]
	Moderately	52.54 [42.69–62.4]	52.61 [48.36–56.86]
	Very much	64.88 [54.73–75.02]	53.62 [45.89–61.34]
	Extremely	58.5 [42.02–74.98]	47.32 [34.72–59.92]
*p*-value		*F*(4,92) = 4.4; *p* < 0.01	*F*(4,280) = 14.55; p < 0.001
EMF-worries			
EMF-sensitivity	Not at all	4.04 [2.39–5.69]	3.15 [2.35–3.94]
	Somewhat	8.67 [6.23–11.1]	7.69 [6.62–8.75]
	Moderately	11.67 [10.14–13.2]	10.24 [9.4–11.08]
	Very much	13.13 [11.82–14.43]	13.12 [12.07–14.16]
	Extremely	13.7 [11.76–15.64]	12.68 [11.09–14.28]
*p*-value		F(4,92) = 18.52; *p* < 0.001	*F*(4,280) = 69.58; *p* < 0.001

P1: Period 1; P2: Period 2.

**Table 4:** EMF source worries vs. perceived EMF-sensitivity, by period (mean [95% CI]).

		P1	P2
Mobile phone-worries			
EMF-sensitivity	Not at all	0.96 [0.53–1.39]	0.86 [0.66–1.07]
	Somewhat	2.1 [1.53–2.66]	1.91 [1.65–2.16]
	Moderately	2.88 [2.46–3.29]	2.61 [2.39–2.83]
	Very much	3.25 [2.91–3.59]	3.53 [3.28–3.78]
	Extremely	3.7 [3.28–4.12]	3.45 [3.05–3.86]
*p*-value		F(4,92) = 20.43; p < 0.001	F(4,280) = 74.78; p < 0.001
5G antennas-worries			
EMF-sensitivity	Not at all	1.19 [0.66–1.72]	0.89 [0.63–1.14]
	Somewhat	2.19 [1.48–2.9]	1.89 [1.56–2.22]
	Moderately	2.92 [2.32–3.51]	2.71 [2.45–2.96]
	Very much	3.31 [2.69–3.93]	3.29 [2.88–3.71]
	Extremely	3.4 [2.61–4.19]	3.09 [2.41–3.78]
*p*-value		F(4,92) = 8.43; p < 0.001	F(4,280) = 38.01; p < 0.001
2-4G antennas-worries			
EMF-sensitivity	Not at all	0.92 [0.48–1.36]	0.77 [0.56–0.99]
	Somewhat	2.14 [1.56–2.73]	1.98 [1.68–2.29]
	Moderately	3.17 [2.76–3.57]	2.57 [2.33–2.81]
	Very much	3.5 [3.19–3.81]	3.50 [3.23–3.77]
	Extremely	4	3.59 [3.17–4.01]
*p*-value		F(4,92) = 27.41; p < 0.001	F(4,280) = 68.03; p < 0.001
High voltage power line-worries			
EMF-sensitivity	Not at all	0.96 [0.59–1.33]	0.63 [0.44–0.81]
	Somewhat	2.24 [1.55–2.92]	1.91 [1.61–2.21]
	Moderately	2.71 [2.29–3.13]	2.35 [2.10–2.61]
	Very much	3.06 [2.5–3.62]	2.79 [2.36–3.22]
	Extremely	2.6 [1.52–3.68]	2.55 [1.86–3.23]
*p*-value		F(4,92) = 9.58; p < 0.001	F(4,280) = 39.40; p < 0.001

P1: Period 1; P2: Period 2.

### 3.2 Hyp 2: the more EMF sensitive people are, the greater their adoption of avoidance strategies

**Figure 2:**
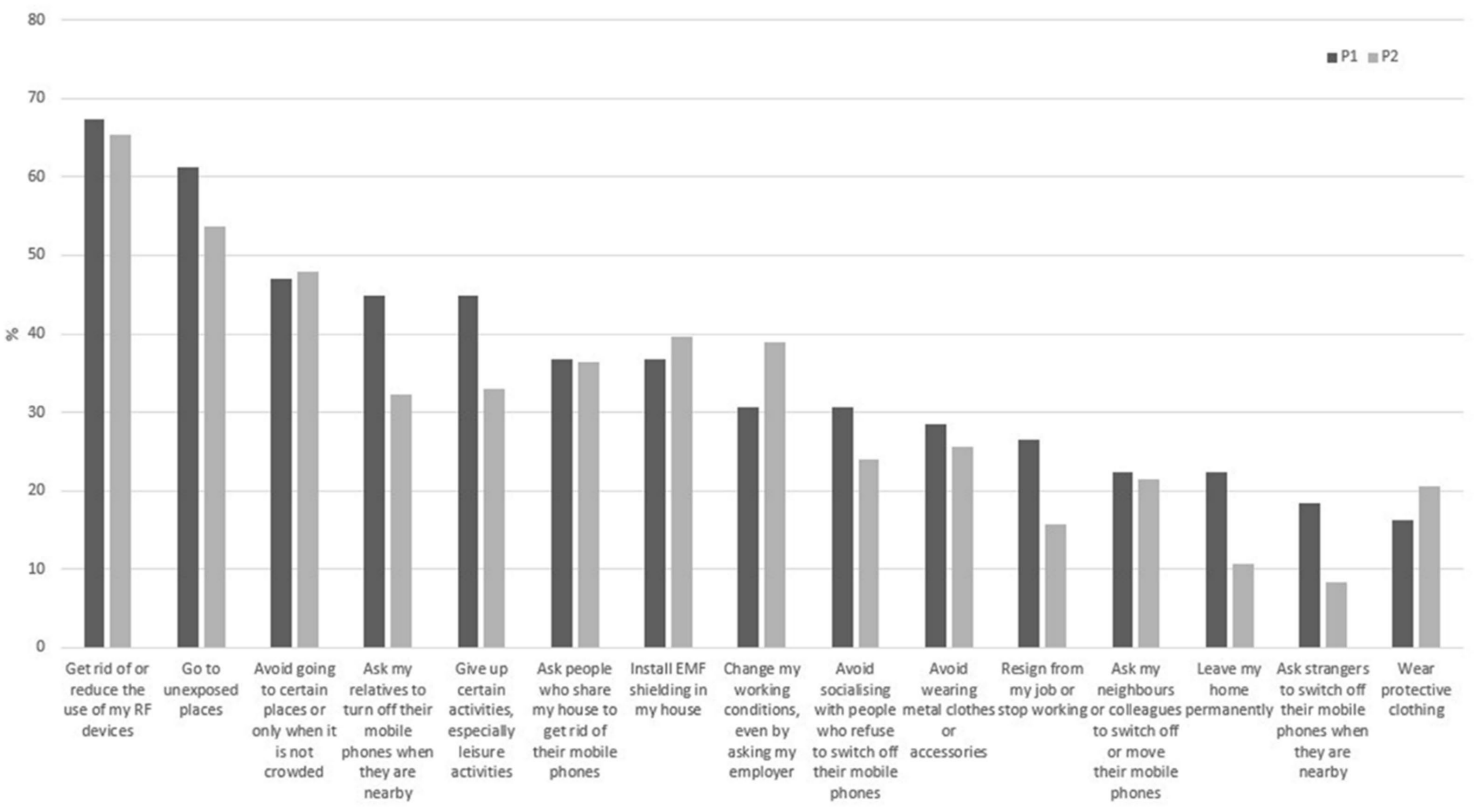
Proportion of respondents who adopted different avoidance strategies at least once a month by period.

In P1, 44.3% of participants reported adopting strategies to limit their exposure at least once in the past month, while 27.3% did so in P2. Regardless of the period, the three most common strategies to avoid exposure were to eliminate or reduce the use of RF devices, to go to places that people considered to be unexposed to recover and recharge, and to avoid going to exposed places or only when there were fewer people (Figure 2).

**Figure 3:**
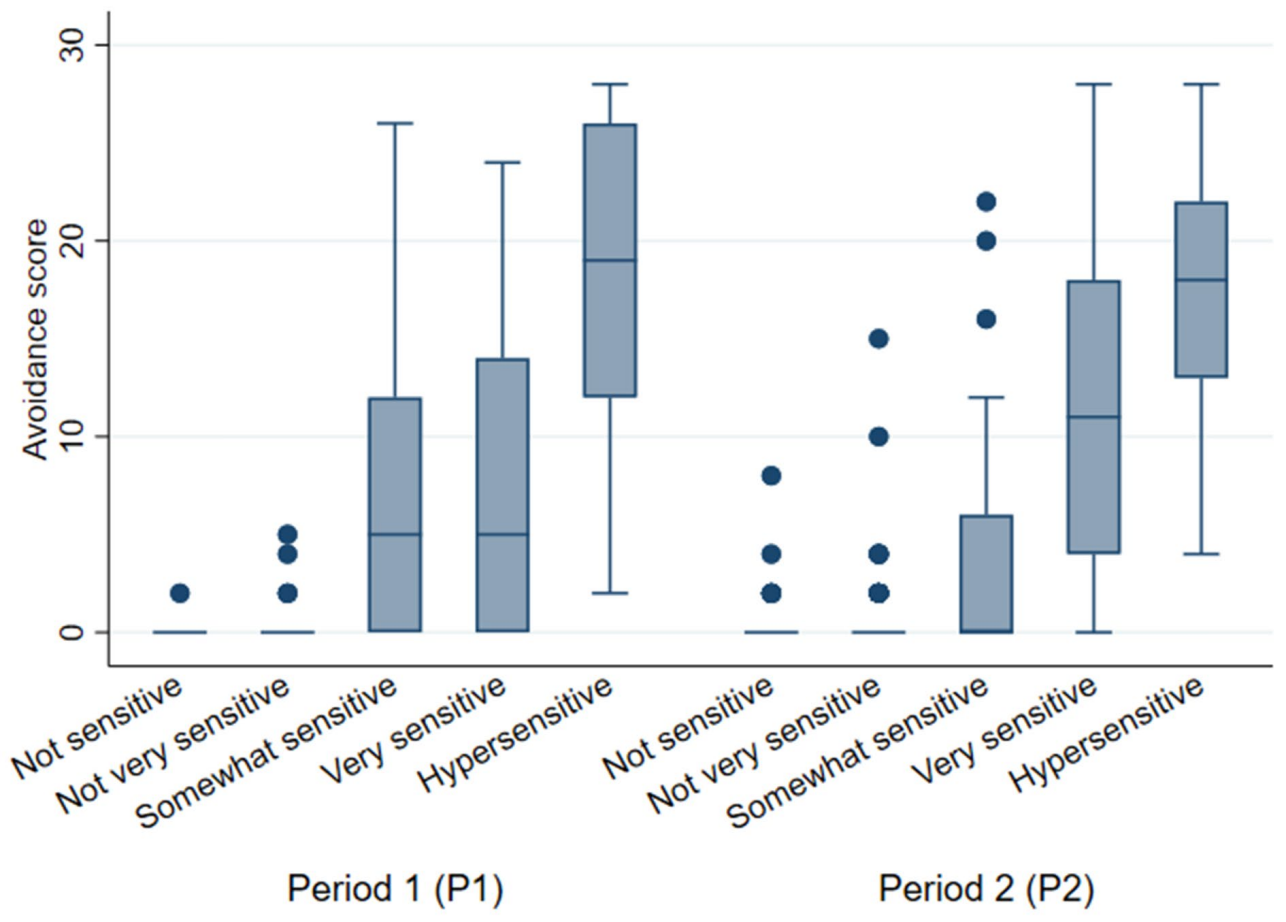
Avoidance score versus EMF sensitivity by period.

The avoidance score was significantly different for the five EMF sensitivity categories in both periods (P1: *F* (4,92) = 20.04; *p* < 0.001; P2: *F* (4,280) = 97.46; *p* < 0.001) (Figure 3). The greater the reported sensitivity to EMFs, the greater the number of avoidance strategies.

### 3.3 Hyp 3: the more people adopt exposure avoidance strategies, the lower their exposure perception to EMF sources

#### 3.3.1 Exposure perception

**Figure 4:**
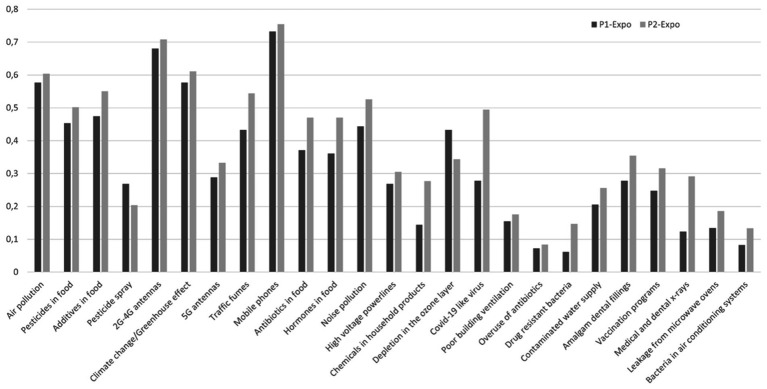
Histogram of MHW exposure perception (from 0 “No” to 1 “Yes, very exposed”) in P1 and P2.

The highest exposure perceptions were observed for mobile phones, 2G-4G antennas and climate change/greenhouse effects, and the lowest were observed for the overuse of antibiotics and bacteria in air conditioning systems, both in P1 and P2 (Figure 4).

Among EMF sources, exposure to mobile phones was generally perceived as high, closely followed by exposure to 2G-4G antennas and then exposure to 5G antennas and high-voltage power lines, both in P1 and P2 (Table 5).

**Table 5:** Scores of exposure perceptions and items related to EMF source exposure in P1 and P2 (means [95% Conf. Interval]).

	P1	P2
MHW_exposure	8.14 [6.95–9.34]	9.64 [8.89–10.39]
noEMF_exposure	6.18 [5.17–7.18]	7.54 [6.90–8.18]
EMF_exposure	1.97 [1.71–2.23]	2.10 [1.95–2.26]
Mobile phone-exposure	0.73 [0.64–0.82]	0.75 [0.70–0.79]
2-4G antennas-exposure	0.68 [0.59–0.77]	0.71 [0.66–0.76]
5G antennas-exposure	0.29 [0.20–0.38]	0.33 [0.28–0.39]
HVPL-exposure	0.27 [0.18–0.36]	0.31 [0.25–0.36]

P1: Period 1; P2: Period 2; MHW: Modern Health Worries; HVPL: High voltage power line.

#### 3.3.2 Exposure perception to EMF sources vs. EMF sensitivity

In both periods, there were differences in the EMF_exposure score according to EMF-sensitivity: an increase in exposure perception was observed as sensitivity increases.

In P1, there was no difference in the distribution of the various levels of EMF-sensitivity of the participants reporting no or high exposure to EMF sources (Table 6). In contrast, in P2, significant differences were found for each source: the most sensitive participants more often reported high exposure than did the non-sensitive respondents. Regardless of the source, the proportion of people in P2 who reported not being sensitive was greater among those who reported not being highly exposed, while the proportion of people who were more sensitive followed an opposite pattern (Table 6).

**Table 6:** Proportion of people reporting being highly or not exposed to EMF (EMF_exposure score) and EMF sources vs. EMF-sensitivity, by period (*p*-values refer to the comparison between the proportion of participants reporting being highly or not exposed to the different EMF sources, in P1 and in P2).

		P1		P2	
		No	Yes	No	Yes
Mobile phone-exposure					
	Categories	*N* = 26	*N* = 71	*N* = 70	*N* = 215
EMF-sensitivity (%)	Not at all	30.8	25.4	55.7	26.5
	Somewhat	34.6	16.9	10	21.9
	Moderately	19.2	26.8	15.7	31.6
	Very much	3.8	21.1	10	12.6
	Extremely	11.5	9.9	8.6	7.4
*p*-value		0.102		<0.001	
5G antennas-exposure					
	Categories	*N* = 69	*N* = 28	*N* = 190	*N* = 95
EMF-sensitivity (%)	Not at all	29.0	21.4	39.5	22.1
	Somewhat	23.2	17.9	22.1	12.6
	Moderately	24.6	25.0	26.8	29.5
	Very much	14.5	21.4	5.8	24.2
	Extremely	8.7	14.3	5.8	11.6
*p*-value		0.749		<0.001	
2-4G antennas-exposure					
	Categories	*N* = 31	*N* = 66	*N* = 83	*N* = 202
EMF-sensitivity (%)	Not at all	32.3	24.2	50.6	26.7
	Somewhat	29.0	18.2	20.5	18.3
	Moderately	22.6	25.8	19.3	21.2
	Very much	12.9	18.2	6	14.4
	Extremely	3.2	13.6	3.6	9.4
*p*-value		0.391		0.001	
High voltage power line-exposure					
	Categories	*N* = 71	*N* = 26	*N* = 198	*N* = 87
EMF-sensitivity (%)	Not at all	29.6	19.2	39.9	19.5
	Somewhat	19.7	26.9	16.7	24.1
	Moderately	25.4	23.1	27.3	28.7
	Very much	15.5	19.2	9.1	18.4
	Extremely	9.9	11.5	7.1	9.2
*p*-value		0.818		0.006	

P1: Period 1; P2: Period 2 – N: number of participants.

### 3.4 Multivariate analyses in relation to hypotheses

#### 3.4.1 Ordered logistic regression

Overall regressions were statistically significant for P1 (LR chi2 (17) = 124.06, *p* < 0.001) and P2 (LR chi2 (18) = 330.98, *p* < 0.001). As shown in Supplementary material S5, for both periods, the analyses indicated that the SymptomScore was associated with EMF-sensitivity, followed by EMF_worries. Age in P1 and living in the Flemish region in P2 had significant impacts on EMF sensitivity. The Avoidance score also played a significant role in both periods. On the other hand, in P2, noEMF_worries was slightly inversely related to EMF sensitivity.

In contrast, other variables, including gender, urbanization, employment, health status, EMF_exposure and noEMF_exposure in both periods, region and noEMF worry in P1, and age in P2, did not significantly predict the rating of EMF sensitivity. However, we must consider this with caution since it is quite complex to consider the direction of the association between worries and sensitivity, and between perception of exposure and avoidance strategies.

#### 3.4.2 GBM analyses

**Figure 5:**
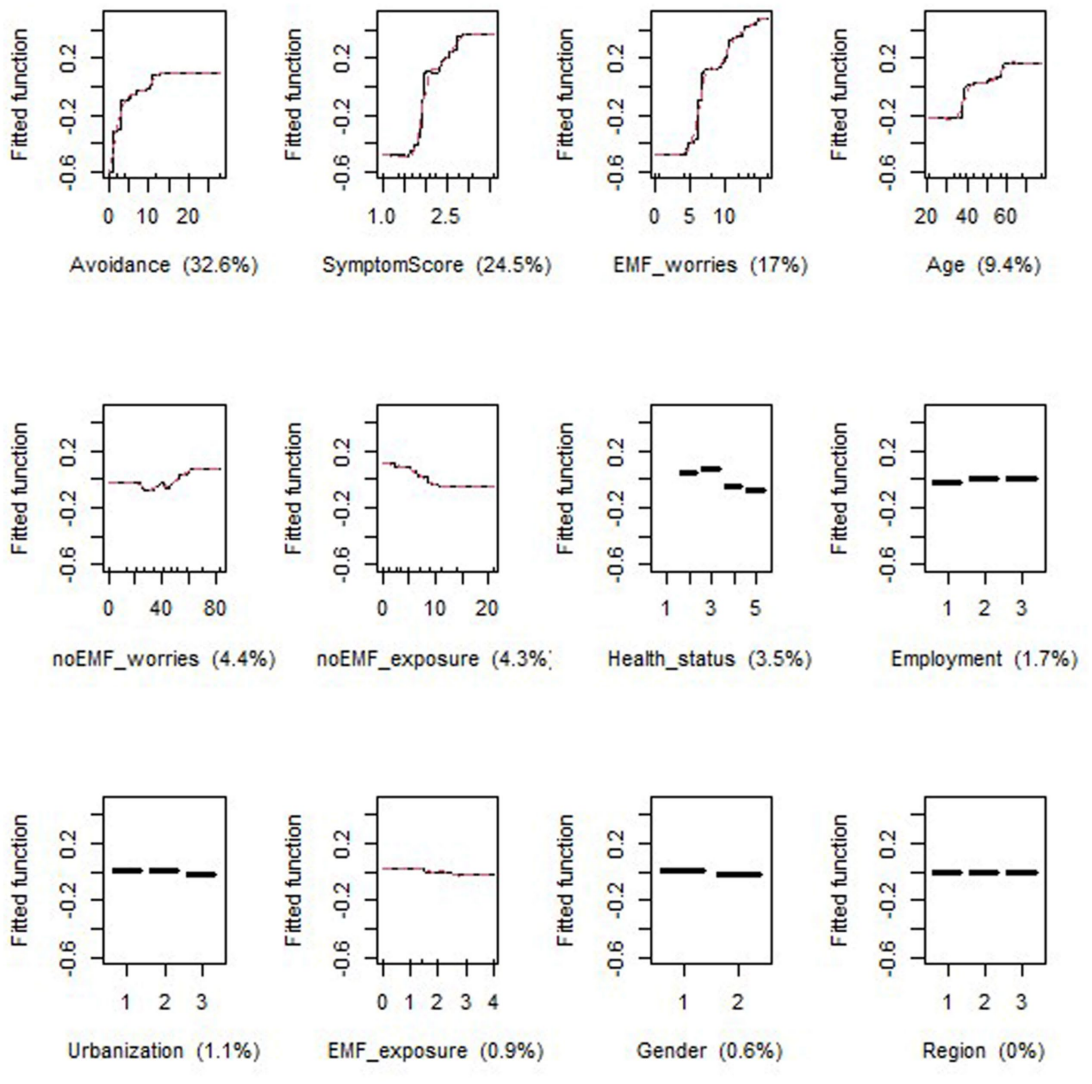
Response curves between EMF sensitivity as the dependent variable and generic, health and specific variables (the latter based on our hypotheses) for period 1 (P1); The Y-axis, labeled ‘Fitted function,’ represents the predicted contribution of each independent variable to the dependent variable, as modeled by the GBM algorithm.

**Figure 6:**
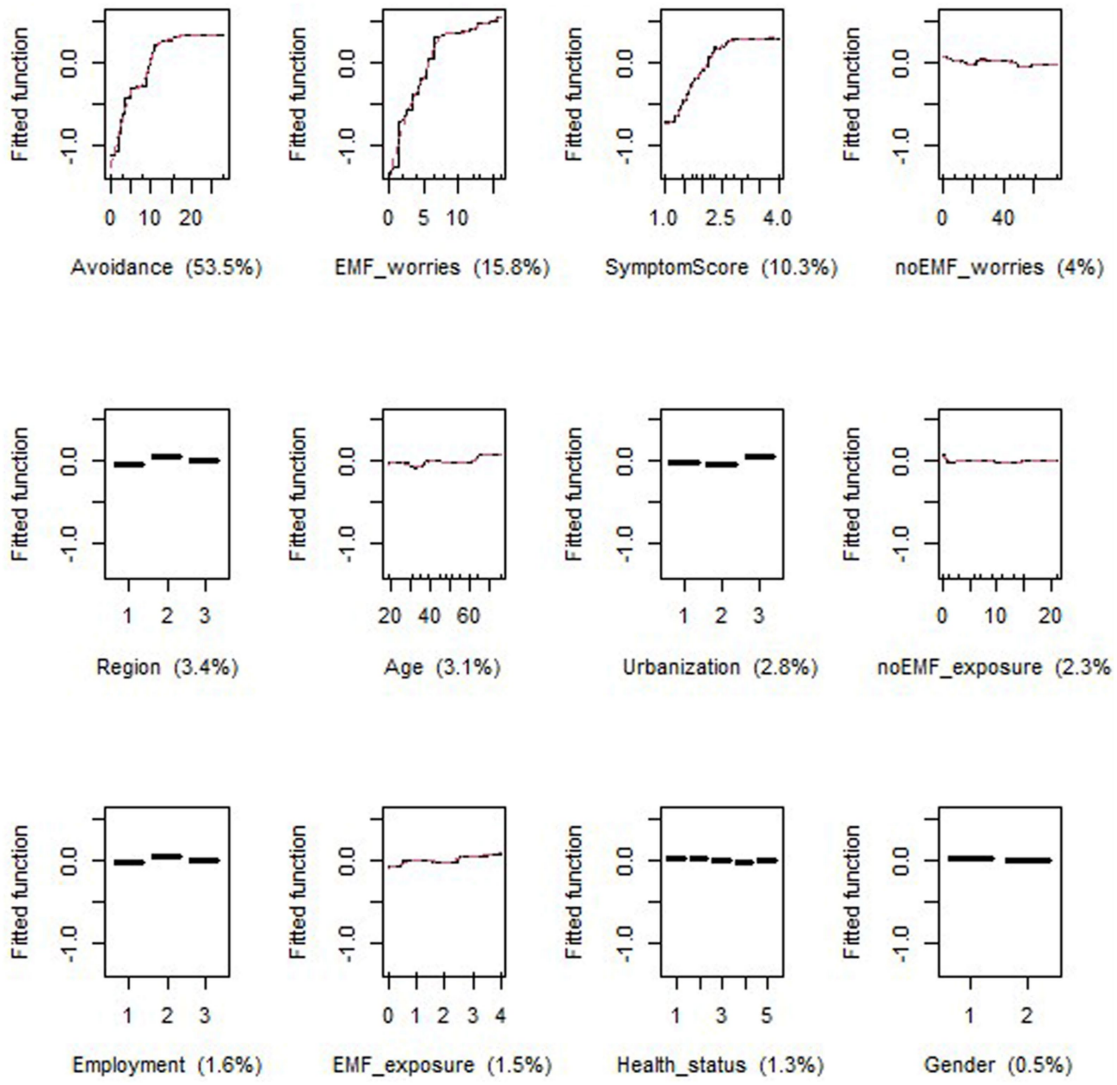
Response curves between EMF sensitivity as the dependent variable and generic, health and specific variables (the latter based on our hypotheses) for period 2 (P2); The Y-axis, labeled ‘Fitted function,’ represents the predicted contribution of each independent variable to the dependent variable, as modeled by the GBM algorithm.

In both periods, the most important variables in the models for EMF-sensitivity were Avoidance score, SymptomScore and EMF_worries, which together accounted for 74.1 and 79.1% of the variance in P1 (Figure 5) and P2 (Figure 6), respectively. However, in P2, Avoidance score was by far the most important variable, accounting for more than half of the model’s explanation. The relationship was quasi-linear in both periods for EMF_worries, while a plateau was reached for Avoidance and EMF_worries (in both periods at scores of approximately 15 and 3, respectively).

In P1, Age accounted for 9.4% of the model, while the variables noEMF_worries, noEMF_exposure and Health_status each accounted for approximately 3–4% (Figure 5). In P2, the same proportions applied to the variables noEMF_worries, Region and Age (Figure 6). The age curve in P1 showed a predicted relationship that increased sharply between the ages of 35 and 40 and then stabilized, whereas this increase was less obvious in P2. The noEMF_worries curve should be interpretated with caution due to the lack of concordance between P1 and P2 analyses.

In both periods, generic variables such as employment, urbanization and gender played a minor role in explaining the model, including region in P1. Among the specific variables, perceived EMF_exposure did not noticeably contribute to the rating of EMF sensitivity in either P1 or P2, not following the response curve of avoidance.

## 4 Discussion

### 4.1 Main findings

The aim of this study was to collect data to improve knowledge of the characteristics of people who report different levels of sensitivity to EMF by assessing their MHW, perception of exposure and avoidance strategies.

The analysis of MHW items showed, contrary to Baliatsas et al. (20) and Bailer et al. (5), that all EMF sources considered, especially 2G-4G antennas, are among the greatest worries. This could be related to the characteristics of our population, which includes a larger proportion of people who consider themselves to be EMF sensitive, as was intended during recruitment.

We observed differences between P1 and P2 regarding the percentages of participants from different regions (with a larger proportion of Flemish participants in P2) and the level of urbanization (consistent with regional proportions) (Supplementary material S4). However, there were no differences in participant characteristics related to health, sensitivity, and symptoms (Table 2) between the two periods.

Our first hypothesis was based on the positive association between reporting higher levels of EMF sensitivity and higher MHW scores, particularly on the EMF items of the MHW scale. The results of this study confirmed this hypothesis.

Considering our second hypothesis, we observed more frequent avoidance strategies for those reporting greater EMF sensitivity.

Finally, regarding our third hypothesis that the adoption of exposure avoidance strategies would lead to lower exposure perceptions of EMF sources, our results suggest that this hypothesis should be rejected. Indeed, despite the more frequent avoidance strategies of those reporting sensitivity, there is no indication of a reduction in the exposure perception among the most EMF sensitive individuals or in relation to the increased EMF sensitivity. This was confirmed in the multivariate analysis, where EMF exposure did not account for EMF exposure in the model. The hypothesis of lower exposure perception is thus not confirmed. However, despite the apparent lack of effect on their perception of being exposed, individuals express setting up exposure avoidance strategies. It is recognized that avoidance strategies can lead to social exclusion, work incapacity and financial difficulties (27), which need to be considered (11).

The use of GBM models provided complementary insights to the ordered logistic regression analyses. For example, GBM analyses highlighted that avoidance strategies accounted for the largest share of variance in EMF sensitivity, particularly in P2, where they represented over 50% of the model’s explanatory power. This finding underscores the central role of behavioural responses in perceived EMF sensitivity. Additionally, the non-linear relationships identified by the GBM models—for instance, the plateau effect observed for the symptom score and avoidance strategies—provided deeper insights into how these factors influence sensitivity at different levels.

### 4.2 How to explain contradictions between exposure perception and avoidance strategies

Our results showed that the most sensitive people perceived themselves to be more exposed than did the other participants. This can be interpreted in two directions.

First, the exposure perception of highly sensitive people could be influenced by their avoidance strategies. To reduce their symptoms and to make their avoidance strategies as effective as possible, they are forced to look for - and in some ways find - what they consider to be the potential origin of these symptoms. By focusing on them, highly sensitive people may become aware of the wide diversity of EMF sources in their surroundings. This could explain why these avoidance strategies, as they are implemented, are not sufficient to reduce their exposure perception. Second, they use avoidance strategies, but these strategies are not effective. Based on Dieudonné (31) and Van den Bergh et al. (32), several potential explanations are possible:

1. Avoidance strategies are ineffective because IEI-EMF people do not know exactly the sources of their symptoms (33). However, they are sufficiently convinced that EMF affect their health; therefore, they adopt ‘random’ adaptive behaviors without seeing the benefits. Indeed, by not knowing exactly what sources of exposure they are sensitive to, strategies may be ineffective in not specifically targeting the actual sources causing their symptoms or the whole of them. Additionally, as their symptoms do not disappear in a perennial way, they may conclude that they are indeed exposed to something without being able to say what. This would explain their high perception of exposure, equally as high as that of less or not sensitive individuals, despite the strategies they put in place;
2. Avoidance strategies are ineffective because these strategies are not systematic. This is not because individuals adopt avoidance strategies that they adopt enough to feel less exposed to. Indeed, since the sources of exposure are multiple and everywhere around them, the strategies in place are not effective, not because of their capacity to prevent the effects of waves but because of the overwhelming multiplicity of sources. In other words, these strategies could be effective if they were systematic;
3. Avoidance strategies could be ineffective because the increased attention people pay to the sources of exposure in their immediate environment could increase short-term nocebo reactions and long-term negative conditioning (32, 34);
4. Avoidance strategies are ineffective because they are disconnected from the real sources of their symptoms, which could be due to something different than the EMF they target as the cause of those symptoms, leading to a mistaken attribution (35).

### 4.3 Limitations of the study

The sample in this study could be a point of concern. Indeed, particularly in P1, the number of respondents, notably the proportion of Flemish citizens, is quite low due to limited access to contact persons or institutions likely to relay survey information during this period. This may limit the generalizability of the findings and their applicability as clinical guidance. Future research should aim for larger and more diverse participant pools to strengthen the reliability of the conclusions.

Moreover, in contrast to previous surveys dealing with MHW and IEI-EMF (20, 24), which included a larger number of participants, the recruitment of individuals was directed primarily towards IEI-EMF people. As a result, there was a greater proportion of people with this profile in this study, which was relevant for the analysis of their behavioral characteristics. Furthermore, this study does not follow a longitudinal design; therefore, it does not allow us to identify a genuine evolution of the sensitivity or of its characteristics, as Traini et al. (36), Martens et al. (37) or Röösli et al. (38).

Our analysis revealed regional differences between the P1 and P2 populations, particularly in terms of levels of urbanization. These differences may influence perceptions of EMF exposure, as urban areas often have visible EMF infrastructure, such as transformers, electricity cables, and mobile phone masts. In fact, ordered logistic regressions showed a significant impact of living in Flanders on the rating of EMF sensitivity in P2, with a higher proportion of Flemish inhabitants. However, this was not linked to the degree of urbanization, as evaluated here based on postal codes. Moreover, GBM analyses did not confirm this. One of the limitations of our study is the lack of data on the visibility of infrastructure, which could affect participants’ perceptions. Future research should include questions on the proximity and visibility of these infrastructures in order to better understand their impact on perception and behavior.

Another limitation that could also be mentioned is the classification of respondents as more or less sensitive to EMFs based on a single question. Indeed, Szemerszky et al. (39) proposed complementing the sensitivity assessment commonly used in surveys with additional questions considering the ratio of symptoms and the impact of sensitivity on people’s lives. More specificity in the definition of EMF sensitive people could have provided more accurate information for comparisons between groups. Finally, multivariate analyses by GBM applied for exploratory purposes, which are still not widely used in this type of analysis, revealed their interests, but adjustments could improve their performance.

### 4.4 For further investigations

We have observed that risk perception and avoidance strategies are important variables in defining the level of sensitivity. However, avoidance strategies have been shown to be ineffective in reducing perceived exposure to EMF sources, while often leading to substantial financial costs and significant social and professional consequences (40). These behaviors, which reflect a response to a perceived threat, can be analysed through the prism of Protection Motivation Theory (PMT) (41), which provides a framework for understanding how individuals assess threats and adapt their protective behaviors. Indeed, four elements are expected to play a role in how individuals are driven to react in a protective way towards a perceived threat, such as NIR and its possible health implications: the *perceived severity* of the hazard and its *likelihood of occurring* decide on their “threat appraisal,” while their sense of being able to cope with the threat, the so-called “coping appraisal,” is influenced by both their *response efficacy* (the belief that the threat can be mitigated) and their perceived *self-efficacy* (the belief in their own ability to take action to mitigate the threat).

On the basis of the PMT, we could hypothesize that self-efficacy and response effectiveness scores are low in these individuals, due to the ubiquity of EMFs (difficult to avoid completely) and the perceived lack of control over exposure. This ineffectiveness of avoidance strategies raises questions about their relevance in reducing perceived exposure and associated symptoms. Our results highlight the need for empirical verification of weak coping appraisals in IEI-CEM individuals to better tailor interventions. It would also be relevant to explore other approaches, such as cognitive-behavioral interventions, to reinforce the feeling of control and self-efficacy (42). Confirming this hypothesis would highlight the need to critically evaluate avoidance strategies and their psychological impact, which could inform more effective interventions for highly sensitive individuals.

Beyond these questions, there is a need to focus on the resources and capacity to improve the quality of life of IEI-EMF people. Whatever the reasons for the ineffectiveness - or very relative effectiveness - of their avoidance strategies to feel less exposed, as described in Section 4.2, their suffering requires effective care to reduce the impact of symptoms on their daily lives. Therefore, despite the limited evidence to date, cognitive behavioral therapies (CBTs) could be an interesting research prospect for developing therapeutic tools (43). Nevertheless, other strategies should also be tested on the basis of new IEI-EMF models, for example, the comprehensive model explaining the onset of symptoms and their link with environmental agents developed by Van den Bergh et al. (32, 44). It may offer new perspectives for helping people with IEI-EMF to cope with their symptoms, but further work is needed to test their validity.

## 5 Conclusion

People who perceive themselves as highly sensitive to EMFs report worries about EMF sources and exposure avoidance behaviors, but exposure perceptions remain high. Given the distress, social isolation and professional difficulties of some of these people, it is necessary to consider the relevance of avoidance strategies.

## Data Availability

The datasets presented in this study can be found in online repositories. The names of the repository/repositories and accession number(s) can be found below: The dataset analysed for this study can be found in the Zenodo repository, doi: 10.5281/zenodo.7858285.

## References

[1] Katsumata S Ichikohji T Nakano S Yamaguchi S Ikuine F. Changes in the use of mobile devices during the crisis: immediate response to the COVID-19 pandemic. Comput Hum Behav Rep. (2022) 5:100168. doi: 10.1016/j.chbr.2022.100168, PMID: 35079660 PMC8769530

[2] Charafeddine R. Cinquième enquête de santé COVID-19. Numéro de dépôt: D/2020/14.440/96. Bruxelles, Belgique: Disponible en ligne. (2020). doi: 10.25608/xcxd-7784

[3] Simkó M Mattsson MO. 5G wireless communication and health effects - a pragmatic review based on available studies regarding 6 to 100 GHz. Int J Environ Res Public Health. (2019) 16:3406. doi: 10.3390/ijerph16183406, PMID: 31540320 PMC6765906

[4] Elzanaty A Chiaraviglio L Alouini M-S. 5G and EMF exposure: misinformation, open questions, and potential solutions. Front Comms Net. (2021) 2:635716. doi: 10.3389/frcmn.2021.635716, PMID: 39897177

[5] Bailer J Witthöft M Rist F. Modern health worries and idiopathic environmental intolerance. J Psychosom Res. (2008) 65:425–33. doi: 10.1016/j.jpsychores.2008.05.006, PMID: 18940372

[6] Kaptein AA Helder DI WChr K Rief W Moss-Morris R Petrie KJ. Modern health worries in medical students. J Psychosom Res. (2005) 58:453–7. doi: 10.1016/j.jpsychores.2004.12.001, PMID: 16026662

[7] Köteles F Simor P Czető M Sárog N Szemerszky R. Modern health worries - the dark side of spirituality? Scand J Psychol. (2016) 57:313–20. doi: 10.1111/sjop.12297, PMID: 27231809

[8] Petrie KJ Sivertsen B Hysing M Broadbent E Moss-Morris R Eriksen HR. Thoroughly modern worries the relationship of worries about modernity to reported symptoms, health and medical care utilization. J Psychosom Res. (2001) 51:395–401. doi: 10.1016/s0022-3999(01)00219-7, PMID: 11448708

[9] Dömötör Z Nordin S Witthöft M Köteles F. Modern health worries: a systematic review. J Psychosom Res. (2019) 124:109781. doi: 10.1016/j.jpsychores.2019.109781, PMID: 31443819

[10] American Academy of Allergy, Asthma and Immunology (AAAAI) Board of Directors. J Allergy Clin Immunol. (1999) 103:36–40. doi: 10.1016/S0091-6749(99)70522-1, PMID: 9893182

[11] Anses. Hypersensibilité électromagnétique ou intolérance environnementale idiopathique attribuée aux champs électromagnétiques (Avis de l’Agence Nationale de Sécurité Sanitaire de l’alimentation, de l’environnement et du Travail, p. 359) [Rapport d’expertise collective]. (2018). Available at: https://www.anses.fr/en/system/files/AP2011SA0150Ra.pdf

[12] World Health Organization. Environmental health criteria 238: Extremely low frequency fields. Geneva: World Health Organization (2007).

[13] Schmiedchen K Driessen S Oftedal G. Methodological limitations in experimental studies on symptom development in individuals with idiopathic environmental intolerance attributed to electromagnetic fields (IEI-EMF) – a systematic review. Environ Health. (2019) 18:88. doi: 10.1186/s12940-019-0519-x, PMID: 31640707 PMC6805477

[14] Oftedal G Straume A Johnsson A Stovner L. Mobile phone headache: a double blind, sham-controlled provocation study. Cephalalgia. (2007) 27:447–55. doi: 10.1111/j.1468-2982.2007.01336.x, PMID: 17359515

[15] Rubin GJ Nieto-Hernandez R Wessely S. Idiopathic environmental intolerance attributed to electromagnetic fields (formerly ‘electromagnetic hypersensitivity’): an updated systematic review of provocation studies. Bioelectromagnetics. (2010) 31:1–11. doi: 10.1002/bem.20536, PMID: 19681059

[16] de Graaff MB Bröer C. ‘We are the canary in a coal mine’: establishing a disease category and a new health risk. Health Risk Soc. (2012) 14:129–47. doi: 10.1080/13698575.2012.661040

[17] Dieudonné M. Does electromagnetic hypersensitivity originate from nocebo responses? Indications from a qualitative study. Bioelectromagnetics. (2016) 37:14–24. doi: 10.1002/bem.21937, PMID: 26369906

[18] Dieudonné M. Becoming electro-hypersensitive: a replication study. Bioelectromagnetics. (2019) 40:188–200. doi: 10.1002/bem.22180, PMID: 30920673

[19] World Health Organization. Constitution of the World Health Organization. Available at: https://apps.who.int/gb/bd/PDF/bd47/EN/constitution-en.pdf?ua=1. (2005). (Accessed November 28, 2024).

[20] Baliatsas C van Kamp I Hooiveld M Lebret E Yzermans J. The relationship of modern health worries to non-specific physical symptoms and perceived environmental sensitivity: a study combining self-reported and general practice data. J Psychosom Res. (2015) 79:355–61. doi: 10.1016/j.jpsychores.2015.09.004, PMID: 26526308

[21] Indregard A-MR Ihlebæk CM Eriksen HR. Modern health worries, subjective health complaints, health care utilization, and sick leave in the Norwegian working population. Int J Behav Med. (2013) 20:371–7. doi: 10.1007/s12529-012-9246-1, PMID: 22729981

[22] Köteles F Simor P. Modern health worries, somatosensory amplification and subjective symptoms: a longitudinal study: a longitudinal study. Int J Behav Med. (2013) 20:38–41. doi: 10.1007/s12529-011-9217-y, PMID: 22207442

[23] Andersen JH Jensen JC. Modern health worries and visits to the general practitioner in a general population sample: an 18 month follow-up study. J Psychosom Res. (2012) 73:264–7. doi: 10.1016/j.jpsychores.2012.07.007, PMID: 22980530

[24] Szemerszky R Dömötör Z Witthöft M Köteles F. Modern health worries and idiopathic environmental intolerance attributed to electromagnetic fields are associated with paranoid ideation. J Psychosom Res. (2021) 146:110501. doi: 10.1016/j.jpsychores.2021.110501, PMID: 33930739

[25] Witthöft M Rubin GJ. Are media warnings about the adverse health effects of modern life self-fulfilling? An experimental study on idiopathic environmental intolerance attributed to electromagnetic fields (IEI-EMF). J Psychosom Res. (2013) 74:206–12. doi: 10.1016/j.jpsychores.2012.12.002, PMID: 23438710

[26] Baliatsas C van Kamp I Kelfkens G Schipper M Bolte J Yzermans J. Non-specific physical symptoms in relation to actual and perceived proximity to mobile phone base stations and powerlines. BMC Public Health. (2011) 11:421. doi: 10.1186/1471-2458-11-421, PMID: 21631930 PMC3118249

[27] Crasson M. L’hypersensibilité à l’électricité: Une approche multidisciplinaire pour un problème multifactoriel. Revue de la littérature. Eur Rev Appl Psychol. (2005) 55:51–67. doi: 10.1016/j.erap.2004.10.001

[28] Eurostat. Correspondence table LAU – NUTS 2021, EU-27 and EFTA / available candidate countries [data set]. Eurostat. Available at: https://ec.europa.eu/eurostat/documents/345175/501971/EU-27-LAU-2021-NUTS-2021.xlsx (2021) (Accessed November 28, 2024).

[29] Elith J Leathwick JR Hastie T. A working guide to boosted regression trees. J Anim Ecol. (2008) 77:802–13. doi: 10.1111/j.1365-2656.2008.01390.x, PMID: 18397250

[30] StatBel. Population density. Available at: https://statbel.fgov.be/en/themes/population/population-density. (Accessed February 19, 2024).

[31] Dieudonné M. Electromagnetic hypersensitivity: a critical review of explanatory hypotheses. Environ Health. (2020) 19:48. doi: 10.1186/s12940-020-00602-0, PMID: 32375774 PMC7201940

[32] Van den Bergh O Brown RJ Petersen S Witthöft M. Idiopathic environmental intolerance: a comprehensive model. Clin Psychol Sci. (2017) 5:551–67. doi: 10.1177/2167702617693327

[33] Bordarie J Dieudonné M Ledent M Prignot N. A qualitative approach to experiential knowledge identified in focus groups aimed at co-designing a provocation test in the study of electrohypersensitivity. Ann Med. (2022) 54:2362–74. doi: 10.1080/07853890.2022.2114605, PMID: 36135790 PMC9518295

[34] Haanes JV Nordin S Hillert L Witthöft M van Kamp I van Thriel C. “Symptoms associated with environmental factors” (SAEF) – towards a paradigm shift regarding “idiopathic environmental intolerance” and related phenomena. J Psychosom Res. (2020) 131:109955. doi: 10.1016/j.jpsychores.2020.109955, PMID: 32058864

[35] Köteles F Szemerszky R Freyler A Bárdos G. Somatosensory amplification as a possible source of subjective symptoms behind modern health worries. Scand J Psychol. (2011) 52:174–8. doi: 10.1111/j.1467-9450.2010.00846.x, PMID: 21029108

[36] Traini E Martens AL Slottje P Vermeulen RCH Huss A. Time course of health complaints attributed to RF-EMF exposure and predictors of electromagnetic hypersensitivity over 10 years in a prospective cohort of Dutch adults. Sci Total Environ. (2023) 856:159240. doi: 10.1016/j.scitotenv.2022.159240, PMID: 36209879

[37] Martens A Slottje P Smid T Kromhout H Vermeulen RCH Timmermans DRM. Longitudinal associations between risk appraisal of base stations for mobile phones, radio or television and non-specific symptoms. J Psychosom Res. (2018) 112:81–9. doi: 10.1016/j.jpsychores.2018.07.008, PMID: 30097140

[38] Röösli M Mohler E Frei P. Sense and sensibility in the context of radiofrequency electromagnetic field exposure. C R Phys. (2010) 11:576–84. doi: 10.1016/j.crhy.2010.10.007

[39] Szemerszky R Dömötör Z Köteles F. One single question is not sufficient to identify individuals with electromagnetic hypersensitivity. CPE. (2019) 1:668. doi: 10.32872/cpe.v1i4.35668

[40] Verrender A Loughran SP Anderson V Hillert L Rubin GJ Oftedal G. IEI-EMF provocation case studies: a novel approach to testing sensitive individuals. Bioelectromagnetics. (2018) 39:132–43. doi: 10.1002/bem.22095, PMID: 29125197

[41] Rogers RW. A protection motivation theory of fear appeals and attitude Change1. J Psychol. (1975) 91:93–114. doi: 10.1080/00223980.1975.9915803, PMID: 28136248

[42] Bandura A. The explanatory and predictive scope of self-efficacy theory. J Soc Clin Psychol. (1986) 4:359–73. doi: 10.1521/jscp.1986.4.3.359

[43] Rubin GJ Hahn G Everitt BS Cleare AJ Wessely S. Are some people sensitive to mobile phone signals? Within participants double blind randomised provocation study. BMJ. (2006) 332:886–91. doi: 10.1136/bmj.38765.519850.55, PMID: 16520326 PMC1440612

[44] Van den Bergh O Bräscher A-K Witthöft M. Idiopathic environmental intolerance: a treatment model. Cogn Behav Pract. (2021) 28:281–92. doi: 10.1016/j.cbpra.2020.05.002

[45] Friedman JH Hastie T Tibshirani R. Additive logistic regression: a statistical view of boosting. Annals Statis. (2000) 28:337–407.

[46] Schapire R. The boosting approach to machine learning – an overview. msri workshop on nonlinear estimation and classification. Denison DD Hansen MH Holmes C Mallick B Yu B editors. New York: Springer. (2003)., PMID:

