# Supplementary material for: Modern health worries and exposure perceptions of individuals reporting varying levels of sensitivity to electromagnetic fields: results of two successive surveys
Source: Front Public Health. 2025 Feb 19;13:1536167. doi: 10.3389/fpubh.2025.1536167 (PMC11879838; doi:10.3389/fpubh.2025.1536167)
Supplement: Supplementary file 1 [file Supplementary_file_1.docx]

Supplementary 1

# List of variables used in the study

| **Variable** | **Description** | **Question** | **Values** | **Domain** |
| --- | --- | --- | --- | --- |
| Age | Year of birth |  | Continuous or by categories:  1: 18-24y (%)  2: 25-44y (%)  3: 45-64y (%)  4: >64y (%) | Demography |
| Gender | Gender |  | 1: Women  2: Men | Demography |
| Region | Region |  | 1: Brussels  2: Flanders  3: Wallonia | Demography |
| Urbanisation | Degree of urbanisation | Based on DEGURBA classification (level 1) | 1: Densely populated urban areas  2: Intermediate density areas  3: Rural, low population density areas | Demography |
| Employment | Employment | Are you currently in paid employment, possibly temporarily interrupted (due to the lockdown) ? | 1: Yes  2: No  3: No answer | Demography |
| SymptomScore | Score of all 10 items from the question on symptoms (average) | During the lockdown, did you suffer from any of the following health problems?  1: Never  2: Less than one time a week  3: Every week or so  4: Every day or almost every day | 1 to 4 | Health status |
| Health_status | Health status evaluation | How would you rate your current health status?  5: Very good  4: Good  3: Fair  2: Poor  1: Very poor | 1 to 5 | Health status |
| MHW | Score of all 25 items of the Modern Health Worries scale | MHW scale: Concerns related to 25 items assessed on a 5-point Likert scale (0:Not at all, 1: Somewhat, 2: Moderately, 3: Very much, 4: Extremely). | 0 to 100 | Risk perception |
| noEMF_worries | Score of all items of the Modern Health Worries scale without items dedicated to EMF | MHW scale: Concerns related to 21 items assessed on a 5-point Likert scale (0:Not at all, 1: Somewhat, 2: Moderately, 3: Very much, 4: Extremely). | 0 to 84 | Risk perception |
| EMF_worries | Score of all items dedicated to EMF in the Modern Health Worries scale | MHW scale: Concerns related to 4 items assessed on a 5-point Likert scale (0:Not at all, 1: Somewhat, 2: Moderately, 3: Very much, 4: Extremely). | 0 to 16 | Risk perception |
| noVaccine_worries | Score of all items of the Modern Health Worries scale without the item related to the vaccination programs | MHW scale: Concerns related to 24 items assessed on a 5-point Likert scale (0:Not at all, 1: Somewhat, 2: Moderately, 3: Very much, 4: Extremely). | 0 to 96 | Risk perception |
| Vaccine_worries | Score of the item | MHW scale: item on concerns related to vaccination programs assessed on a 5-point Likert scale (0:Not at all, 1: Somewhat, 2: Moderately, 3: Very much, 4: Extremely). | 0 to 4 | Risk perception |
| MHW_exposure | Score of all items of the MHW perceived exposure | Secondary scale to the MHW scale | 0 to 25 | Exposure perception |
| noEMF_exposure | Score of all items of the MHW perceived exposure without items dedicated to EMF | Secondary scale to the MHW scale | 0 to 21 | Exposure perception |
| EMF_exposure | Score of the 4 items dedicated to EMF exposure in the Modern Health Worries scale | Secondary scale to the MHW scale | 0 to 4 | Exposure perception |
| Avoidance | Score of all items related to *exposure avoidance*  strategies | Sum of exposure reduction strategies | 0 to 30 | Coping |
| EMF-sensitivity | EMF sensitivity evaluation | How would you rate your current EMF sensitivity? | 0: Not sensitive  1: Not very sensitive  2: Somewhat sensitive  3: Very sensitive  4: Hypersensitive | Electrohyper-sensitivity |
