# Supplementary material for: Modern health worries and exposure perceptions of individuals reporting varying levels of sensitivity to electromagnetic fields: results of two successive surveys
Source: Front Public Health. 2025 Feb 19;13:1536167. doi: 10.3389/fpubh.2025.1536167 (PMC11879838; doi:10.3389/fpubh.2025.1536167)
Supplement: Supplementary file 2 [file Supplementary_file_2.docx]

Supplementary 2

# The Modern Health Worries scale

*Note: In French and Dutch in the surveys*

Please rate how concerned you are about the impact of the following agents on your health.

|  | Not at all | Somewhat | Moderately | Very much | Extremely |  | Are you VERY exposed to this agent |
| --- | --- | --- | --- | --- | --- | --- | --- |
| Poor building ventilation | € | € | € | € | € |  |  Yes   No |
| Contaminated water supply | € | € | € | € | € |  |  Yes   No |
| Vaccination programs | € | € | € | € | € |  |  Yes   No |
| Overuse of antibiotics | € | € | € | € | € |  |  Yes   No |
| Chemicals in household products | € | € | € | € | € |  |  Yes   No |
| Leakage from microwave ovens | € | € | € | € | € |  |  Yes   No |
| Bacteria in air conditioning systems | € | € | € | € | € |  |  Yes   No |
| Drug resistant bacteria | € | € | € | € | € |  |  Yes   No |
| Amalgam dental fillings | € | € | € | € | € |  |  Yes   No |
| Medical and dental x-rays | € | € | € | € | € |  |  Yes   No |
| Air pollution | € | € | € | € | € |  |  Yes   No |
| Noise pollution | € | € | € | € | € |  |  Yes   No |
| Depletion in the ozone layer | € | € | € | € | € |  |  Yes   No |
| Traffic fumes | € | € | € | € | € |  |  Yes   No |
| Pesticide spray | € | € | € | € | € |  |  Yes   No |
| Additives in food | € | € | € | € | € |  |  Yes   No |
| Pesticides in food | € | € | € | € | € |  |  Yes   No |
| Antibiotics in food | € | € | € | € | € |  |  Yes   No |
| Hormones in food | € | € | € | € | € |  |  Yes   No |
| Mobile phones | € | € | € | € | € |  |  Yes   No |
| 5G antennas | € | € | € | € | € |  |  Yes   No |
| Mobile phone antennas (2G-3G-4G) | € | € | € | € | € |  |  Yes   No |
| High voltage powerlines | € | € | € | € | € |  |  Yes   No |
| Climate change/Greenhouse effect | € | € | € | € | € |  |  Yes   No |
| Covid-19 like virus | € | € | € | € | € |  |  Yes   No |
| Others :  ……………………... | € | € | € | € | € |  |  Yes   No |
