# Supplementary material for: Modern health worries and exposure perceptions of individuals reporting varying levels of sensitivity to electromagnetic fields: results of two successive surveys
Source: Front Public Health. 2025 Feb 19;13:1536167. doi: 10.3389/fpubh.2025.1536167 (PMC11879838; doi:10.3389/fpubh.2025.1536167)
Supplement: Supplementary file 3 [file Supplementary_file_3.docx]

Supplementary 3

1. **Exposure avoidance strategies**

*Note 1:* *In French and Dutch in the surveys*

*Note 2: The question 'Have you ever tried to reduce your exposure to electromagnetic fields? (Yes/No)' from the survey is intended to clarify the context for the subsequent question, which specifically refers to actions taken to reduce EMF exposure.*

What actions have you taken (regardless of their success)? (Several answers possible)

| *Actions taken…* | No | Yes, since last month | Yes, over a month ago |
| --- | --- | --- | --- |
| I've got rid of my radio-emitting equipment, use it less or adapted it (for example, I've thrown away my mobile phone, only use it to send text messages, fitted it with an anti-wave tablet, etc.). | □ | □ | □ |
| I've asked the people who share my home to get rid of their radio transmitters. | □ | □ | □ |
| I have asked my neighbours or work colleagues to turn off or move away any equipment that is bothering me (mobile or DECT phones, internet boxes, etc.). | □ | □ | □ |
| I ask my friends and family to turn off their electronic devices when they spend time with me. | □ | □ | □ |
| I ask people I don't know to turn off their phones when I'm nearby. | □ | □ | □ |
| J’évite de fréquenter certaines personnes, en particulier celles qui refusent d’éteindre leurs appareils. | □ | □ | □ |
| I avoid certain places or frequent them only at certain times (public transport, shops, etc.). | □ | □ | □ |
| I've given up certain activities, particularly leisure activities. | □ | □ | □ |
| I go for occasional or regular refreshments in protected areas^[[1]](#footnote-1)^ | □ | □ | □ |
| I wear protective clothing (hat, cap, shawl, jacket, etc.). | □ | □ | □ |
| I avoid wearing clothes or accessories with metal (glasses, jewellery, belts, bras, etc.). | □ | □ | □ |
| I have modified my workstation or my working conditions, asking my employer if necessary (deactivating Wi-Fi on the premises, installing a shielded computer, reducing business travel, etc.). | □ | □ | □ |
| I have installed protection against electromagnetic fields in my home (canopies, curtains, paint, etc.) or modified my electrical installation. | □ | □ | □ |
| I've left my job or stopped working (without necessarily finding or starting a new one). | □ | □ | □ |
| I have left my home permanently (moved house, moved in with relatives, wandered, etc.). | □ | □ | □ |

1. In the context of these surveys, 'protected areas' refer to locations perceived by individuals as having reduced EMF exposure, such as remote natural environments. [↑](#footnote-ref-1)
