# Supplementary material for: Modern health worries and exposure perceptions of individuals reporting varying levels of sensitivity to electromagnetic fields: results of two successive surveys
Source: Front Public Health. 2025 Feb 19;13:1536167. doi: 10.3389/fpubh.2025.1536167 (PMC11879838; doi:10.3389/fpubh.2025.1536167)
Supplement: Supplementary file 4 [file Supplementary_file_4.docx]

Supplementary 4

1. **General characteristics of the participants included and excluded because of missing data on sensitivity and MHW**

|  | **P1** | | | **P2** | | |  |
| --- | --- | --- | --- | --- | --- | --- | --- |
|  | **Included** | **Excluded** | **Incl/Excl** | **Included** | **Excluded** | **Incl/Excl** | **P1/P2**  **(included)** |
|  | n=97 | n=56 | p-value | n=285 | n=161 | p-value | p-value |
| **Age (%)** | | | | | | | |
| 18-24y | 3.1 | 0 | 0.274 | 2.8 | 3.1 | 0.168 | 0.336 |
| 25-44y | 42.3 | 55.4 |  | 36.1 | 44.1 |  |  |
| 45-64y | 41.2 | 30.4 |  | 51.2 | 40.4 |  |  |
| >64y | 13.4 | 14.3 |  | 9.8 | 12.4 |  |  |
| Missing data | / | / |  | / | / |  |  |
| **Gender (%)** | | | | | | | |
| Female | 57.5 | 62.5 | 0.611 | 52.6 | 64.6 | 0.037 | 0.637 |
| Male | 42.5 | 37.5 |  | 47.4 | 35.4 |  |  |
| **Region (%)** | | | | | | | |
| Brussels | 9.3 | 19.6 | 0.153 | 26.0 | 24.2 | 0.454 | <0.001 |
| Flanders | 2.1 | 1.8 |  | 29.5 | 35.4 |  |  |
| Wallonia | 88.7 | 78.6 |  | 44.2 | 40.4 |  |  |
| (Missing) |  |  |  | 0.3 |  |  |  |
| **Urbanization level (%)** | | | | | | | |
| Densely populated urban areas | 44.3 | 48.2 | 0.930 | 41.4 | 36.0 | 0.447 | 0.001 |
| Intermediate density areas | 26.8 | 25 |  | 43.5 | 49.7 |  |  |
| Rural, low population density areas | 29.9 | 26.8 |  | 14.7 | 14.3 |  |  |
| (Missing) |  |  |  | 0.3 |  |  |  |
| **Employment (%)** | | | | | | | |
| Yes | 69.1 | 58.9 | 0.143 | 73.3 | 65.2 | 0.101 | 0.568 |
| No | 26.8 | 28.6 |  | 21.8 | 25.5 |  |  |
| No answer | 4.1 | 12.5 |  | 4.9 | 9.3 |  |  |

*Legend: P1: Period 1, P2: Period 2*
