# Supplementary material for: Modern health worries and exposure perceptions of individuals reporting varying levels of sensitivity to electromagnetic fields: results of two successive surveys
Source: Front Public Health. 2025 Feb 19;13:1536167. doi: 10.3389/fpubh.2025.1536167 (PMC11879838; doi:10.3389/fpubh.2025.1536167)
Supplement: Supplementary file 5 [file Supplementary_file_5.docx]

Supplementary 5

# Coefficients of ordered logistic regression of the model for EMF sensitivity by generic, health and specific variables (the latter based on our hypotheses), by period

|  | **P1** | | | | | **P2** | | | | |
| --- | --- | --- | --- | --- | --- | --- | --- | --- | --- | --- |
| Independent variables | Coefficient | 95% confidence interval | | z | p > \|z\| | Coefficient | 95% confidence interval | | z | p > \|z\| |
| Age | .05 | .01 | .10 | 2.20 | * | .00 | -.02 | .03 | 0.39 | ns |
| Women.Gender: *Men* | .67 | -1.68 | .339 | -1.30 | ns | .08 | -.43 | .60 | 0.31 | ns |
| Urban.Urbanization: *Intermediate density areas Rural, low population density areas* | .06 -.58 | -1.14 -1.70 | 1.25 .54 | 0.09 -1.02 | ns ns | -.54 .21 | -1.29 -.71 | .21 1.14 | -1.42 0.45 | ns ns |
| Brussels.Region: *Flanders Wallonia* | -3.73 -.98 | -7.79 -2.96 | .34 1.01 | -1.80 -.96 | ns ns | 1.06 .28 | .12 -.57 | 1.99 1.14 | 2.21 0.65 | * ns |
| Yes.Employment: *No No answer* | -.00 -.09 | -1.33 -2.41 | 1.32 2.24 | -0.00 -0.07 | ns ns | .41 -.44 | -.26 -1.64 | 1.08 .77 | 1.19 -0.71 | ns ns |
| X.Health_status: *Poor*  *Fair Good Very good* | 0 1.26 .30 .24 | 0 -.59 -1.73 -2.04 | 0 3.11 2.33 2.51 | 0 1.33 0.29 0.20 | ns ns ns | 1.44 1.79 1.97 2.04 | -1.55 -1.06 -.93 -.95 | 4.43 4.64 4.88 5.04 | 0.94 1.23 1.33 1.34 | ns ns ns ns |
| SymptomScore | 1.43 | .33 | 2.54 | 2.54 | * | 1.32 | .77 | 1.87 | 4.72 | *** |
| EMF_worries | .33 | .18 | .49 | 4.18 | *** | .34 | .25 | .43 | 7.61 | *** |
| noEMF_worries | -.01 | -.04 | .02 | -0.87 | ns | -.03 | -.05 | -.01 | -2.85 | ** |
| EMF_exposure | -.23 | -.75 | .29 | -0.85 | ns | .19 | -.09 | .46 | 1.33 | ns |
| noEMF_exposure | -.08 | -.20 | .04 | -1.27 | ns | -.04 | -.10 | .03 | -1.10 | ns |
| Avoidance | .14 | .06 | .22 | 3.28 | ** | .20 | .14 | .26 | 6.66 | *** |

*Legend: (1) X.Health_status: X=Poor in P1 and Very poor in P2; (2) *** p<0.001, ** p<.01, * p<.05, ns not significant*
